# (*E*)-1-{4-[Bis(4-meth­oxy­phen­yl)meth­yl]piperazin-1-yl}-3-(3,4-dieth­oxy­phen­yl)prop-2-en-1-one ethanol disolvate

**DOI:** 10.1107/S1600536811055796

**Published:** 2012-01-07

**Authors:** Yan Zhong, XiaoPing Zhang, Bin Wu

**Affiliations:** a School of Chemistry and Chemical Engineering, Southeast University, Sipailou No. 2 Nanjing, Nanjing 210096, People’s Republic of China; b Centre of Laboratory Animals, Nanjing medical University, Hanzhong Road No. 140 Nanjing, Nanjing 210029, People’s Republic of China; c School of Pharmacy, Nanjing Medical University, Hanzhong Road No. 140 Nanjing, Nanjing 210029, People’s Republic of China

## Abstract

The asymmetric unit of the title compound, C_32_H_38_N_2_O_5_·2C_2_H_6_O, contains one main mol­ecule and two solvent mol­ecules, which inter­act *via* inter­molecular O—H⋯O hydrogen bonds. The piperazine ring adopts a chair conformation. The crystal packing exhibits weak inter­molecular C—H⋯O hydrogen bonds and voids of 31 Å^3^.

## Related literature

For the crystal structures of related cinnamic acid derivatives, see: Teng *et al.* (2011 ▶); Zhong & Wu (2011 ▶). For further synthetic details, see: Wu *et al.* (2008 ▶).
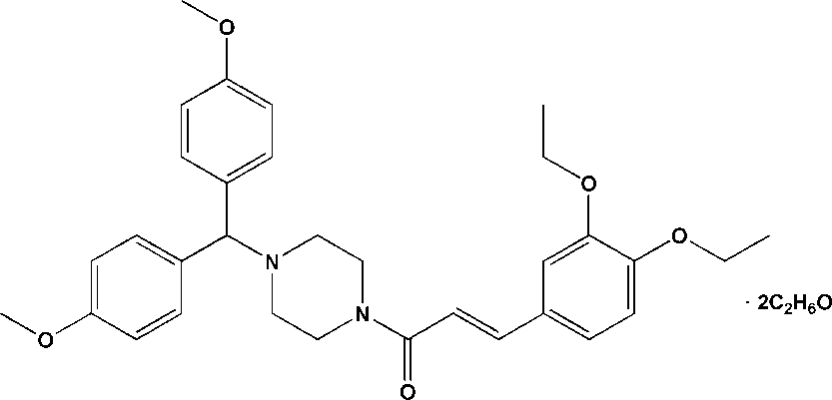


## Experimental

### 

#### Crystal data

C_32_H_38_N_2_O_5_·2C_2_H_6_O
*M* *_r_* = 622.78
Triclinic, 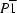

*a* = 12.511 (3) Å
*b* = 12.564 (3) Å
*c* = 13.601 (3) Å
α = 88.13 (3)°
β = 70.62 (3)°
γ = 65.84 (3)°
*V* = 1826.9 (6) Å^3^
*Z* = 2
Mo *K*α radiation
μ = 0.08 mm^−1^
*T* = 293 K
0.30 × 0.10 × 0.10 mm

#### Data collection

Enraf–Nonius CAD-4 diffractometer
Absorption correction: ψ scan (North *et al.*, 1968 ▶) *T*_min_ = 0.977, *T*_max_ = 0.992
6687 measured reflections
6687 independent reflections
2904 reflections with *I* > 2σ(*I*)
3 standard reflections every 200 reflections intensity decay: 1%

#### Refinement

*R*[*F* ^2^ > 2σ(*F* ^2^)] = 0.070
*wR*(*F* ^2^) = 0.166
*S* = 1.00
6687 reflections
412 parameters
2 restraints
H-atom parameters constrained
Δρ_max_ = 0.19 e Å^−3^
Δρ_min_ = −0.16 e Å^−3^

### 

Data collection: *CAD-4 EXPRESS* (Enraf–Nonius, 1989 ▶); cell refinement: *CAD-4 EXPRESS*; data reduction: *XCAD4* (Harms & Wocadlo,1995 ▶); program(s) used to solve structure: *SHELXS97* (Sheldrick, 2008 ▶); program(s) used to refine structure: *SHELXL97* (Sheldrick, 2008 ▶); molecular graphics: *SHELXTL* (Sheldrick, 2008 ▶); software used to prepare material for publication: *SHELXL97*.

## Supplementary Material

Crystal structure: contains datablock(s) I, global. DOI: 10.1107/S1600536811055796/cv5226sup1.cif

Structure factors: contains datablock(s) I. DOI: 10.1107/S1600536811055796/cv5226Isup2.hkl

Supplementary material file. DOI: 10.1107/S1600536811055796/cv5226Isup3.cml

Additional supplementary materials:  crystallographic information; 3D view; checkCIF report

## Figures and Tables

**Table 1:** Hydrogen-bond geometry (Å, °)

*D*—H⋯*A*	*D*—H	H⋯*A*	*D*⋯*A*	*D*—H⋯*A*
O6—H6*A*⋯O3	0.82	1.97	2.699 (4)	147
O7—H7*C*⋯O6	0.82	1.91	2.730 (6)	178
C14—H14*A*⋯O7^i^	0.97	2.54	3.436 (7)	153
C32—H32*A*⋯O4^ii^	0.96	2.57	3.258 (6)	129

Symmetry codes: (i) 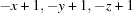; (ii) 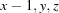.

**Fig. 1.**
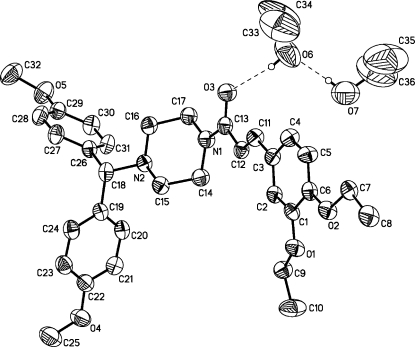
The content of asymmetric part of the title compound showing the atomic numbering and 30% probability displacement ellipsoids. H atoms not involved in hydrogen-bonding (dashed lines) omitted for clarity.

## supplementary crystallographic information

### Comment

As a continuation of our study of cinnamic acid derivatives (Teng *et al.*, 2011; Zhong *et al.*, 2011), we present here the title compound (I).

In (I) (Fig. 1), all bond lengths and angles are normal and correspond to those observed in related compounds (Teng *et al.*, 2011; Zhong *et al.*, 2011). The molecule of (I) exists an E configulation with respect to the C11=C12 ethene bond [1.306 (4)]. The piperazine ring adopts a chair conformation. In the crystal structure, the molecules are linked by intermolecular C—H···O and O—H···O hydrogen bonds (Table 1).

### Experimental

The synthesis follows the method of Wu *et al.* (2008). The title compound was prepared by stirring a mixture of (*E*)-3-(3,4-diethoxyphenyl)acrylic acid (0.945 g; 4 mmol), thionyl chloride (2 ml) and dichloromethane (30 ml) for 6 h at room temperature. The solvent was removed under reduced pressure. The residue was dissolved in acetone (15 ml) and reacted with 1-(bis(4-methoxyphenyl)methyl)iperazine (1.874 g; 6 mmol) in the presence of triethylamine (5 ml) for 12 h at room temperature. The resultant mixture was cooled. The solid, (*E*)-1-(4-(bis(4-methoxyphenyl)methyl)piperazin-1-yl) -3-(3,4-diethoxyphenyl)prop-2-en-1-one obtained was filtered and was recrystallized from ethanol. The pale-yellow single crystals of the title compound used in *x*-ray diffraction studies were grown in ethyl acetate: hexane (2:1) by a slow evaporation at room temperature.

### Refinement

All hydrogen atoms were positioned geometrically (C—H 0.93 - 0.98 Å, O—H 0.82 Å) and refined as riding, with *U*ĩso~(H) = 1.2 or 1.5*U*~eq~ of the carrier atom.

### Crystal data

**Table:**

C_32_H_38_N_2_O_5_·2C_2_H_6_O	*Z* = 2
*M**_r_* = 622.78	*F*(000) = 672
Triclinic, *P*1	*D*_x_ = 1.132 Mg m^−^^3^
*a* = 12.511 (3) Å	Mo *K*α radiation, λ = 0.71073 Å
*b* = 12.564 (3) Å	Cell parameters from 25 reflections
*c* = 13.601 (3) Å	θ = 10–13°
α = 88.13 (3)°	µ = 0.08 mm^−^^1^
β = 70.62 (3)°	*T* = 293 K
γ = 65.84 (3)°	Block, pale-yellow
*V* = 1826.9 (6) Å^3^	0.30 × 0.10 × 0.10 mm

### Data collection

**Table:**

Enraf–Nonius CAD-4 diffractometer	2904 reflections with *I* > 2σ(*I*)
Radiation source: fine-focus sealed tube	*R*_int_ = 0.0000
graphite	θ_max_ = 25.4°, θ_min_ = 1.6°
ω/2θ scans	*h* = −13→15
Absorption correction: ψ scan (North *et al.*, 1968)	*k* = −15→15
*T*_min_ = 0.977, *T*_max_ = 0.992	*l* = 0→16
6687 measured reflections	3 standard reflections every 200 reflections
6687 independent reflections	intensity decay: 1%

### Refinement

**Table:**

Refinement on *F*^2^	Primary atom site location: structure-invariant direct methods
Least-squares matrix: full	Secondary atom site location: difference Fourier map
*R*[*F*^2^ > 2σ(*F*^2^)] = 0.070	Hydrogen site location: inferred from neighbouring sites
*wR*(*F*^2^) = 0.166	H-atom parameters constrained
*S* = 1.00	*w* = 1/[σ^2^(*F*_o_^2^) + (0.064*P*)^2^] where *P* = (*F*_o_^2^ + 2*F*_c_^2^)/3
6687 reflections	(Δ/σ)_max_ < 0.001
412 parameters	Δρ_max_ = 0.19 e Å^−^^3^
2 restraints	Δρ_min_ = −0.15 e Å^−^^3^

### Special details

**Table:**

Geometry. All e.s.d.'s (except the e.s.d. in the dihedral angle between two l.s. planes) are estimated using the full covariance matrix. The cell e.s.d.'s are taken into account individually in the estimation of e.s.d.'s in distances, angles and torsion angles; correlations between e.s.d.'s in cell parameters are only used when they are defined by crystal symmetry. An approximate (isotropic) treatment of cell e.s.d.'s is used for estimating e.s.d.'s involving l.s. planes.
Refinement. Refinement of *F*^2^ against ALL reflections. The weighted *R*-factor *wR* and goodness of fit *S* are based on *F*^2^, conventional *R*-factors *R* are based on *F*, with *F* set to zero for negative *F*^2^. The threshold expression of *F*^2^ > σ(*F*^2^) is used only for calculating *R*-factors(gt) *etc*. and is not relevant to the choice of reflections for refinement. *R*-factors based on *F*^2^ are statistically about twice as large as those based on *F*, and *R*- factors based on ALL data will be even larger.

### Fractional atomic coordinates and isotropic or equivalent isotropic displacement parameters (Å^2^)

**Table:**

	*x*	*y*	*z*	*U*_iso_*/*U*_eq_	
O1	0.7645 (2)	0.43579 (19)	0.73201 (16)	0.0748 (7)	
N1	0.3880 (2)	0.3238 (2)	0.4502 (2)	0.0679 (7)	
C1	0.6388 (3)	0.4767 (3)	0.7589 (2)	0.0600 (8)	
O2	0.6341 (2)	0.58409 (19)	0.90097 (16)	0.0763 (7)	
N2	0.4710 (2)	0.1072 (2)	0.32428 (18)	0.0589 (7)	
C2	0.5791 (3)	0.4492 (3)	0.7042 (2)	0.0604 (9)	
H2A	0.6264	0.3976	0.6426	0.073*	
O3	0.2255 (2)	0.4493 (2)	0.58302 (18)	0.0793 (7)	
C3	0.4490 (3)	0.4953 (3)	0.7369 (2)	0.0620 (9)	
O4	1.0072 (2)	−0.2941 (2)	0.0568 (2)	0.0970 (8)	
C4	0.3823 (3)	0.5725 (3)	0.8292 (2)	0.0747 (10)	
H4A	0.2954	0.6051	0.8532	0.090*	
O5	0.2255 (3)	−0.0921 (2)	0.0774 (2)	0.1021 (9)	
C5	0.4408 (3)	0.6019 (3)	0.8857 (2)	0.0757 (10)	
H5A	0.3933	0.6527	0.9477	0.091*	
C6	0.5677 (3)	0.5574 (3)	0.8519 (2)	0.0652 (9)	
C7	0.5645 (3)	0.6754 (3)	0.9877 (2)	0.0774 (11)	
H7A	0.5113	0.6515	1.0447	0.093*	
H7B	0.5119	0.7464	0.9663	0.093*	
C8	0.6542 (3)	0.6980 (3)	1.0227 (3)	0.0998 (13)	
H8A	0.6094	0.7638	1.0766	0.150*	
H8B	0.7109	0.7152	0.9643	0.150*	
H8C	0.7003	0.6298	1.0500	0.150*	
C9	0.8405 (3)	0.3706 (3)	0.6294 (2)	0.0761 (10)	
H9A	0.8141	0.4160	0.5760	0.091*	
H9B	0.8323	0.2975	0.6254	0.091*	
C10	0.9728 (3)	0.3462 (4)	0.6129 (3)	0.1238 (17)	
H10A	1.0259	0.3027	0.5450	0.186*	
H10B	0.9979	0.3011	0.6661	0.186*	
H10C	0.9798	0.4191	0.6169	0.186*	
C11	0.3817 (3)	0.4704 (3)	0.6783 (2)	0.0689 (9)	
H11A	0.2956	0.5000	0.7119	0.083*	
C12	0.4248 (3)	0.4126 (3)	0.5852 (2)	0.0629 (9)	
H12A	0.5102	0.3808	0.5474	0.076*	
C13	0.3408 (4)	0.3975 (3)	0.5399 (3)	0.0627 (9)	
C14	0.5202 (3)	0.2540 (3)	0.3899 (3)	0.0734 (10)	
H14A	0.5711	0.2641	0.4261	0.088*	
H14B	0.5415	0.2812	0.3216	0.088*	
C15	0.5478 (3)	0.1252 (3)	0.3761 (2)	0.0661 (9)	
H15A	0.6354	0.0804	0.3346	0.079*	
H15B	0.5318	0.0967	0.4442	0.079*	
C16	0.3399 (3)	0.1738 (3)	0.3898 (2)	0.0673 (9)	
H16A	0.3243	0.1451	0.4578	0.081*	
H16B	0.2868	0.1618	0.3572	0.081*	
C17	0.3071 (3)	0.3040 (3)	0.4043 (3)	0.0696 (9)	
H17A	0.3165	0.3343	0.3369	0.084*	
H17B	0.2206	0.3458	0.4498	0.084*	
C18	0.5014 (3)	−0.0179 (3)	0.3039 (2)	0.0615 (9)	
H18A	0.4825	−0.0482	0.3719	0.074*	
C19	0.6393 (3)	−0.0892 (3)	0.2416 (2)	0.0572 (8)	
C20	0.7021 (3)	−0.0505 (3)	0.1564 (2)	0.0639 (9)	
H20A	0.6619	0.0237	0.1384	0.077*	
C21	0.8259 (3)	−0.1216 (3)	0.0964 (3)	0.0719 (10)	
H21A	0.8682	−0.0942	0.0391	0.086*	
C22	0.8859 (4)	−0.2317 (3)	0.1212 (3)	0.0714 (10)	
C23	0.8234 (4)	−0.2700 (3)	0.2074 (3)	0.0784 (10)	
H23A	0.8638	−0.3439	0.2258	0.094*	
C24	0.7014 (4)	−0.1997 (3)	0.2664 (3)	0.0726 (10)	
H24A	0.6598	−0.2270	0.3243	0.087*	
C25	1.0723 (4)	−0.4088 (4)	0.0787 (3)	0.1177 (15)	
H25A	1.1550	−0.4439	0.0271	0.177*	
H25B	1.0284	−0.4555	0.0768	0.177*	
H25C	1.0778	−0.4048	0.1471	0.177*	
C26	0.4215 (3)	−0.0353 (3)	0.2477 (2)	0.0575 (8)	
C27	0.3708 (3)	−0.1137 (3)	0.2767 (3)	0.0730 (10)	
H27A	0.3807	−0.1537	0.3341	0.088*	
C28	0.3052 (3)	−0.1346 (3)	0.2223 (3)	0.0828 (11)	
H28A	0.2718	−0.1892	0.2426	0.099*	
C29	0.2886 (3)	−0.0757 (3)	0.1386 (3)	0.0689 (9)	
C30	0.3385 (3)	0.0033 (3)	0.1085 (3)	0.0718 (10)	
H30A	0.3272	0.0436	0.0516	0.086*	
C31	0.4052 (3)	0.0238 (3)	0.1617 (2)	0.0660 (9)	
H31A	0.4397	0.0773	0.1403	0.079*	
C32	0.1610 (4)	−0.1626 (4)	0.1114 (3)	0.1240 (16)	
H32A	0.1211	−0.1660	0.0630	0.186*	
H32B	0.0990	−0.1298	0.1798	0.186*	
H32C	0.2188	−0.2405	0.1148	0.186*	
O6	0.0879 (4)	0.6828 (3)	0.6384 (3)	0.1925 (19)	
H6A	0.1356	0.6162	0.6429	0.289*	
C33	−0.0052 (9)	0.6966 (9)	0.8023 (7)	0.307 (6)	
H33A	−0.0769	0.7214	0.8657	0.461*	
H33B	0.0398	0.7435	0.7999	0.461*	
H33C	0.0483	0.6155	0.8007	0.461*	
C34	−0.0443 (9)	0.7107 (8)	0.7151 (8)	0.283 (6)	
H34C	−0.1044	0.7900	0.7156	0.340*	
H34D	−0.0751	0.6540	0.7052	0.340*	
O7	0.2275 (4)	0.7986 (4)	0.5355 (3)	0.1730 (16)	
H7C	0.1836	0.7656	0.5666	0.259*	
C36	0.1491 (9)	0.9230 (10)	0.5390 (7)	0.310 (6)	
H36A	0.1965	0.9620	0.4943	0.372*	
H36B	0.0786	0.9342	0.5180	0.372*	
C35	0.1096 (9)	0.9642 (10)	0.6458 (9)	0.406 (9)	
H35A	0.1206	1.0347	0.6516	0.609*	
H35B	0.1583	0.9054	0.6794	0.609*	
H35C	0.0229	0.9806	0.6792	0.609*	

### Atomic displacement parameters (Å^2^)

**Table:**

	*U*^11^	*U*^22^	*U*^33^	*U*^12^	*U*^13^	*U*^23^
O1	0.0722 (16)	0.0763 (16)	0.0590 (14)	−0.0177 (13)	−0.0172 (12)	−0.0220 (12)
N1	0.0720 (19)	0.0664 (18)	0.0625 (17)	−0.0264 (16)	−0.0218 (15)	−0.0141 (14)
C1	0.072 (2)	0.051 (2)	0.0495 (19)	−0.0178 (18)	−0.0207 (18)	−0.0059 (15)
O2	0.0844 (16)	0.0748 (16)	0.0606 (14)	−0.0240 (13)	−0.0236 (12)	−0.0231 (12)
N2	0.0703 (18)	0.0586 (17)	0.0517 (15)	−0.0273 (14)	−0.0253 (14)	−0.0014 (13)
C2	0.072 (2)	0.058 (2)	0.0466 (18)	−0.0269 (18)	−0.0147 (17)	−0.0075 (15)
O3	0.0741 (17)	0.0825 (17)	0.0757 (16)	−0.0296 (14)	−0.0218 (13)	−0.0128 (13)
C3	0.071 (2)	0.064 (2)	0.049 (2)	−0.0329 (19)	−0.0129 (17)	−0.0043 (16)
O4	0.0734 (18)	0.0837 (19)	0.115 (2)	−0.0163 (15)	−0.0312 (16)	0.0002 (16)
C4	0.077 (2)	0.090 (3)	0.053 (2)	−0.038 (2)	−0.0138 (18)	−0.0091 (18)
O5	0.119 (2)	0.119 (2)	0.107 (2)	−0.0741 (19)	−0.0543 (18)	0.0085 (17)
C5	0.066 (3)	0.093 (3)	0.052 (2)	−0.029 (2)	−0.0045 (18)	−0.0272 (19)
C6	0.085 (3)	0.061 (2)	0.0486 (19)	−0.031 (2)	−0.0210 (19)	−0.0049 (16)
C7	0.092 (3)	0.069 (2)	0.064 (2)	−0.031 (2)	−0.0183 (19)	−0.0250 (18)
C8	0.109 (3)	0.094 (3)	0.094 (3)	−0.034 (2)	−0.040 (2)	−0.034 (2)
C9	0.073 (3)	0.083 (3)	0.061 (2)	−0.031 (2)	−0.0113 (18)	−0.0150 (18)
C10	0.072 (3)	0.164 (4)	0.107 (3)	−0.034 (3)	−0.013 (2)	−0.044 (3)
C11	0.076 (2)	0.076 (2)	0.056 (2)	−0.0347 (19)	−0.0203 (18)	−0.0037 (18)
C12	0.073 (2)	0.061 (2)	0.057 (2)	−0.0326 (18)	−0.0198 (18)	−0.0003 (16)
C13	0.075 (2)	0.060 (2)	0.056 (2)	−0.033 (2)	−0.0224 (19)	0.0031 (17)
C14	0.071 (2)	0.075 (2)	0.070 (2)	−0.030 (2)	−0.0188 (19)	−0.0130 (19)
C15	0.077 (2)	0.067 (2)	0.054 (2)	−0.0280 (19)	−0.0236 (18)	−0.0077 (16)
C16	0.077 (2)	0.069 (2)	0.064 (2)	−0.034 (2)	−0.0300 (19)	−0.0003 (18)
C17	0.073 (2)	0.068 (2)	0.070 (2)	−0.0294 (19)	−0.0264 (19)	−0.0051 (18)
C18	0.090 (3)	0.062 (2)	0.0441 (18)	−0.0403 (19)	−0.0271 (18)	0.0134 (15)
C19	0.073 (2)	0.055 (2)	0.0463 (19)	−0.0253 (18)	−0.0251 (17)	0.0044 (16)
C20	0.087 (3)	0.059 (2)	0.058 (2)	−0.034 (2)	−0.035 (2)	0.0094 (17)
C21	0.081 (3)	0.076 (3)	0.066 (2)	−0.038 (2)	−0.028 (2)	0.011 (2)
C22	0.071 (3)	0.065 (2)	0.083 (3)	−0.024 (2)	−0.038 (2)	0.000 (2)
C23	0.085 (3)	0.065 (2)	0.092 (3)	−0.027 (2)	−0.045 (2)	0.013 (2)
C24	0.089 (3)	0.064 (2)	0.072 (2)	−0.032 (2)	−0.038 (2)	0.0120 (19)
C25	0.093 (3)	0.093 (3)	0.138 (4)	−0.012 (3)	−0.039 (3)	0.001 (3)
C26	0.071 (2)	0.055 (2)	0.0467 (18)	−0.0272 (17)	−0.0194 (16)	0.0001 (15)
C27	0.094 (3)	0.072 (2)	0.066 (2)	−0.045 (2)	−0.032 (2)	0.0218 (18)
C28	0.091 (3)	0.080 (3)	0.092 (3)	−0.053 (2)	−0.028 (2)	0.013 (2)
C29	0.074 (2)	0.067 (2)	0.073 (2)	−0.034 (2)	−0.029 (2)	0.0026 (19)
C30	0.093 (3)	0.077 (2)	0.061 (2)	−0.045 (2)	−0.036 (2)	0.0139 (18)
C31	0.090 (3)	0.069 (2)	0.060 (2)	−0.050 (2)	−0.0307 (19)	0.0155 (17)
C32	0.115 (4)	0.148 (4)	0.134 (4)	−0.086 (4)	−0.034 (3)	−0.004 (3)
O6	0.199 (4)	0.113 (3)	0.144 (3)	−0.018 (3)	0.029 (3)	−0.019 (2)
C33	0.248 (11)	0.302 (12)	0.253 (12)	−0.032 (9)	−0.051 (9)	0.022 (10)
C34	0.199 (9)	0.263 (10)	0.248 (11)	0.059 (7)	−0.098 (8)	−0.100 (9)
O7	0.131 (3)	0.178 (4)	0.194 (4)	−0.052 (3)	−0.050 (3)	−0.017 (3)
C36	0.260 (12)	0.305 (14)	0.204 (10)	−0.022 (10)	−0.010 (8)	0.022 (10)
C35	0.219 (10)	0.438 (18)	0.440 (19)	−0.089 (11)	−0.006 (12)	−0.233 (16)

### Geometric parameters (Å, °)

**Table:**

O1—C1	1.357 (3)	C16—H16B	0.9700
O1—C9	1.441 (3)	C17—H17A	0.9700
N1—C13	1.358 (4)	C17—H17B	0.9700
N1—C17	1.453 (4)	C18—C19	1.520 (4)
N1—C14	1.461 (4)	C18—C26	1.525 (4)
C1—C2	1.352 (4)	C18—H18A	0.9800
C1—C6	1.420 (4)	C19—C20	1.366 (4)
O2—C6	1.363 (4)	C19—C24	1.380 (4)
O2—C7	1.434 (3)	C20—C21	1.393 (4)
N2—C15	1.452 (3)	C20—H20A	0.9300
N2—C16	1.462 (4)	C21—C22	1.371 (4)
N2—C18	1.470 (4)	C21—H21A	0.9300
C2—C3	1.396 (4)	C22—C23	1.371 (5)
C2—H2A	0.9300	C23—C24	1.373 (4)
O3—C13	1.243 (4)	C23—H23A	0.9300
C3—C4	1.391 (4)	C24—H24A	0.9300
C3—C11	1.455 (4)	C25—H25A	0.9600
O4—C22	1.369 (4)	C25—H25B	0.9600
O4—C25	1.415 (4)	C25—H25C	0.9600
C4—C5	1.370 (4)	C26—C27	1.360 (4)
C4—H4A	0.9300	C26—C31	1.393 (4)
O5—C29	1.392 (4)	C27—C28	1.372 (4)
O5—C32	1.401 (4)	C27—H27A	0.9300
C5—C6	1.360 (4)	C28—C29	1.364 (5)
C5—H5A	0.9300	C28—H28A	0.9300
C7—C8	1.480 (4)	C29—C30	1.362 (4)
C7—H7A	0.9700	C30—C31	1.368 (4)
C7—H7B	0.9700	C30—H30A	0.9300
C8—H8A	0.9600	C31—H31A	0.9300
C8—H8B	0.9600	C32—H32A	0.9600
C8—H8C	0.9600	C32—H32B	0.9600
C9—C10	1.493 (4)	C32—H32C	0.9600
C9—H9A	0.9700	O6—C34	1.533 (10)
C9—H9B	0.9700	O6—H6A	0.8200
C10—H10A	0.9600	C33—C34	1.405 (7)
C10—H10B	0.9600	C33—H33A	0.9600
C10—H10C	0.9600	C33—H33B	0.9600
C11—C12	1.306 (4)	C33—H33C	0.9600
C11—H11A	0.9300	C34—H34C	0.9700
C12—C13	1.456 (4)	C34—H34D	0.9700
C12—H12A	0.9300	O7—C36	1.459 (10)
C14—C15	1.514 (4)	O7—H7C	0.8200
C14—H14A	0.9700	C36—C35	1.409 (8)
C14—H14B	0.9700	C36—H36A	0.9700
C15—H15A	0.9700	C36—H36B	0.9700
C15—H15B	0.9700	C35—H35A	0.9600
C16—C17	1.518 (4)	C35—H35B	0.9600
C16—H16A	0.9700	C35—H35C	0.9600
			
C1—O1—C9	117.0 (2)	C16—C17—H17B	109.6
C13—N1—C17	121.6 (3)	H17A—C17—H17B	108.1
C13—N1—C14	126.5 (3)	N2—C18—C19	112.0 (3)
C17—N1—C14	111.9 (2)	N2—C18—C26	111.4 (3)
C2—C1—O1	125.7 (3)	C19—C18—C26	110.1 (2)
C2—C1—C6	119.4 (3)	N2—C18—H18A	107.7
O1—C1—C6	114.8 (3)	C19—C18—H18A	107.7
C6—O2—C7	117.1 (3)	C26—C18—H18A	107.7
C15—N2—C16	108.0 (2)	C20—C19—C24	118.5 (3)
C15—N2—C18	112.1 (2)	C20—C19—C18	121.8 (3)
C16—N2—C18	111.6 (2)	C24—C19—C18	119.6 (3)
C1—C2—C3	122.4 (3)	C19—C20—C21	120.3 (3)
C1—C2—H2A	118.8	C19—C20—H20A	119.8
C3—C2—H2A	118.8	C21—C20—H20A	119.8
C4—C3—C2	116.7 (3)	C22—C21—C20	120.5 (3)
C4—C3—C11	119.5 (3)	C22—C21—H21A	119.7
C2—C3—C11	123.8 (3)	C20—C21—H21A	119.7
C22—O4—C25	117.5 (3)	O4—C22—C21	115.6 (4)
C5—C4—C3	121.9 (3)	O4—C22—C23	125.3 (4)
C5—C4—H4A	119.0	C21—C22—C23	119.1 (4)
C3—C4—H4A	119.0	C22—C23—C24	120.2 (4)
C29—O5—C32	117.8 (3)	C22—C23—H23A	119.9
C6—C5—C4	120.6 (3)	C24—C23—H23A	119.9
C6—C5—H5A	119.7	C23—C24—C19	121.3 (4)
C4—C5—H5A	119.7	C23—C24—H24A	119.4
C5—C6—O2	124.9 (3)	C19—C24—H24A	119.4
C5—C6—C1	118.9 (3)	O4—C25—H25A	109.5
O2—C6—C1	116.2 (3)	O4—C25—H25B	109.5
O2—C7—C8	108.3 (3)	H25A—C25—H25B	109.5
O2—C7—H7A	110.0	O4—C25—H25C	109.5
C8—C7—H7A	110.0	H25A—C25—H25C	109.5
O2—C7—H7B	110.0	H25B—C25—H25C	109.5
C8—C7—H7B	110.0	C27—C26—C31	118.5 (3)
H7A—C7—H7B	108.4	C27—C26—C18	120.8 (3)
C7—C8—H8A	109.5	C31—C26—C18	120.6 (3)
C7—C8—H8B	109.5	C26—C27—C28	120.9 (3)
H8A—C8—H8B	109.5	C26—C27—H27A	119.6
C7—C8—H8C	109.5	C28—C27—H27A	119.6
H8A—C8—H8C	109.5	C29—C28—C27	120.3 (3)
H8B—C8—H8C	109.5	C29—C28—H28A	119.9
O1—C9—C10	107.5 (3)	C27—C28—H28A	119.9
O1—C9—H9A	110.2	C30—C29—C28	119.7 (3)
C10—C9—H9A	110.2	C30—C29—O5	115.6 (3)
O1—C9—H9B	110.2	C28—C29—O5	124.7 (3)
C10—C9—H9B	110.2	C29—C30—C31	120.3 (3)
H9A—C9—H9B	108.5	C29—C30—H30A	119.8
C9—C10—H10A	109.5	C31—C30—H30A	119.8
C9—C10—H10B	109.5	C30—C31—C26	120.3 (3)
H10A—C10—H10B	109.5	C30—C31—H31A	119.9
C9—C10—H10C	109.5	C26—C31—H31A	119.9
H10A—C10—H10C	109.5	O5—C32—H32A	109.5
H10B—C10—H10C	109.5	O5—C32—H32B	109.5
C12—C11—C3	129.4 (3)	H32A—C32—H32B	109.5
C12—C11—H11A	115.3	O5—C32—H32C	109.5
C3—C11—H11A	115.3	H32A—C32—H32C	109.5
C11—C12—C13	120.8 (3)	H32B—C32—H32C	109.5
C11—C12—H12A	119.6	C34—O6—H6A	109.5
C13—C12—H12A	119.6	C34—C33—H33A	109.5
O3—C13—N1	118.7 (3)	C34—C33—H33B	109.5
O3—C13—C12	121.2 (3)	H33A—C33—H33B	109.5
N1—C13—C12	120.0 (3)	C34—C33—H33C	109.5
N1—C14—C15	110.5 (3)	H33A—C33—H33C	109.5
N1—C14—H14A	109.5	H33B—C33—H33C	109.5
C15—C14—H14A	109.5	C33—C34—O6	92.2 (8)
N1—C14—H14B	109.5	C33—C34—H34C	113.3
C15—C14—H14B	109.5	O6—C34—H34C	113.3
H14A—C14—H14B	108.1	C33—C34—H34D	113.3
N2—C15—C14	111.0 (3)	O6—C34—H34D	113.3
N2—C15—H15A	109.4	H34C—C34—H34D	110.6
C14—C15—H15A	109.4	C36—O7—H7C	109.5
N2—C15—H15B	109.4	C35—C36—O7	102.2 (9)
C14—C15—H15B	109.4	C35—C36—H36A	111.3
H15A—C15—H15B	108.0	O7—C36—H36A	111.3
N2—C16—C17	111.2 (3)	C35—C36—H36B	111.3
N2—C16—H16A	109.4	O7—C36—H36B	111.3
C17—C16—H16A	109.4	H36A—C36—H36B	109.2
N2—C16—H16B	109.4	C36—C35—H35A	109.5
C17—C16—H16B	109.4	C36—C35—H35B	109.5
H16A—C16—H16B	108.0	H35A—C35—H35B	109.5
N1—C17—C16	110.3 (3)	C36—C35—H35C	109.5
N1—C17—H17A	109.6	H35A—C35—H35C	109.5
C16—C17—H17A	109.6	H35B—C35—H35C	109.5
N1—C17—H17B	109.6		
			
C9—O1—C1—C2	9.1 (5)	N2—C16—C17—N1	−57.4 (3)
C9—O1—C1—C6	−169.3 (3)	C15—N2—C18—C19	−55.2 (3)
O1—C1—C2—C3	−179.8 (3)	C16—N2—C18—C19	−176.5 (2)
C6—C1—C2—C3	−1.5 (5)	C15—N2—C18—C26	−179.0 (3)
C1—C2—C3—C4	0.4 (5)	C16—N2—C18—C26	59.7 (3)
C1—C2—C3—C11	178.0 (3)	N2—C18—C19—C20	−44.4 (4)
C2—C3—C4—C5	−0.2 (5)	C26—C18—C19—C20	80.2 (4)
C11—C3—C4—C5	−177.9 (3)	N2—C18—C19—C24	140.3 (3)
C3—C4—C5—C6	1.1 (6)	C26—C18—C19—C24	−95.1 (3)
C4—C5—C6—O2	178.0 (3)	C24—C19—C20—C21	0.0 (4)
C4—C5—C6—C1	−2.2 (5)	C18—C19—C20—C21	−175.3 (3)
C7—O2—C6—C5	−7.3 (5)	C19—C20—C21—C22	0.8 (5)
C7—O2—C6—C1	172.9 (3)	C25—O4—C22—C21	−179.2 (3)
C2—C1—C6—C5	2.4 (5)	C25—O4—C22—C23	2.0 (5)
O1—C1—C6—C5	−179.2 (3)	C20—C21—C22—O4	179.7 (3)
C2—C1—C6—O2	−177.8 (3)	C20—C21—C22—C23	−1.4 (5)
O1—C1—C6—O2	0.6 (4)	O4—C22—C23—C24	−179.9 (3)
C6—O2—C7—C8	−174.7 (3)	C21—C22—C23—C24	1.3 (5)
C1—O1—C9—C10	174.0 (3)	C22—C23—C24—C19	−0.6 (5)
C4—C3—C11—C12	171.2 (3)	C20—C19—C24—C23	−0.1 (5)
C2—C3—C11—C12	−6.3 (6)	C18—C19—C24—C23	175.3 (3)
C3—C11—C12—C13	−180.0 (3)	N2—C18—C26—C27	−137.3 (3)
C17—N1—C13—O3	−0.3 (5)	C19—C18—C26—C27	97.8 (4)
C14—N1—C13—O3	−178.8 (3)	N2—C18—C26—C31	46.5 (4)
C17—N1—C13—C12	178.3 (3)	C19—C18—C26—C31	−78.3 (4)
C14—N1—C13—C12	−0.2 (5)	C31—C26—C27—C28	0.2 (5)
C11—C12—C13—O3	7.8 (5)	C18—C26—C27—C28	−176.1 (3)
C11—C12—C13—N1	−170.8 (3)	C26—C27—C28—C29	−0.7 (5)
C13—N1—C14—C15	124.4 (3)	C27—C28—C29—C30	0.6 (5)
C17—N1—C14—C15	−54.2 (4)	C27—C28—C29—O5	178.8 (3)
C16—N2—C15—C14	−60.2 (3)	C32—O5—C29—C30	−173.3 (3)
C18—N2—C15—C14	176.4 (3)	C32—O5—C29—C28	8.4 (5)
N1—C14—C15—N2	57.9 (3)	C28—C29—C30—C31	0.1 (5)
C15—N2—C16—C17	60.1 (3)	O5—C29—C30—C31	−178.3 (3)
C18—N2—C16—C17	−176.2 (2)	C29—C30—C31—C26	−0.6 (5)
C13—N1—C17—C16	−124.8 (3)	C27—C26—C31—C30	0.5 (5)
C14—N1—C17—C16	53.9 (4)	C18—C26—C31—C30	176.8 (3)

### Hydrogen-bond geometry (Å, °)

**Table:**

*D*—H···*A*	*D*—H	H···*A*	*D*···*A*	*D*—H···*A*
O6—H6A···O3	0.82	1.97	2.699 (4)	147
O7—H7C···O6	0.82	1.91	2.730 (6)	178
C14—H14A···O7^i^	0.97	2.54	3.436 (7)	153
C32—H32A···O4^ii^	0.96	2.57	3.258 (6)	129

Symmetry codes: (i) −*x*+1, −*y*+1, −*z*+1; (ii) *x*−1, *y*, *z*.
